# A Systematic Review of the Effects of Equol (Soy Metabolite) on Breast Cancer

**DOI:** 10.3390/molecules26041105

**Published:** 2021-02-19

**Authors:** Rafidah Hod, Sandra Maniam, Nurul Huda Mohd Nor

**Affiliations:** Department of Human Anatomy, Faculty of Medicine and Health Sciences, University Putra Malaysia, Serdang 43400, Malaysia; sandra@upm.edu.my (S.M.); hudamohdnor@upm.edu.my (N.H.M.N.)

**Keywords:** soy, isoflavones, breast cancer, in vitro, in vivo, MCF-7 cells

## Abstract

Equol is a soy isoflavone metabolite that can be produced by intestinal bacteria. It is lipophilic and resembles natural oestrogens with an affinity to oestrogen receptors. This review is focused on how equol affects breast cancer, as evidenced by in vivo and in vitro studies. Equol is considered chemoprotective in specific endocrine-related pathologies, such as breast cancer, prostate cancer, cardiovascular diseases, and menopausal symptoms. In humans, not everyone can produce equol from gut metabolism. It is postulated that equol producers benefit more than non-equol producers for all the endocrine-related effects. Equol exists in two enantiomers of *R*-equol and *S*-equol. Earlier studies, however, did not specify which enantiomer was being used. This review considers equol’s type and concentration variations, pathways affected, and its outcome in in vivo and in vitro studies.

## 1. Introduction

Daidzein and genistein are isoflavone phytoestrogen, commonly found in soy foods, such as soy milk, fermented soy or tempeh, miso soup, and tofu [1,2,3]. Having a similar chemical structure to mammalian oestrogens allows daidzein and genistein to exert oestrogenic effects in humans. Studies reporting isoflavones oestrogen receptor binding affinity have classified them as natural selective oestrogen receptor modulators (SERMs) [4]. SERMs exhibit both agonist and antagonist effects, which are tissue-dependent. Soy isoflavone consumption varies among countries. Lower daily dietary soy isoflavone of 0.8 mg [5] and 7–9 mg [6] are reported in Finland and UK, respectively, while higher levels of 97 mg were observed in China [7] and 39.5 mg in Japan [8]. Daidzein is metabolised into equol and *O*-desmethylangolensin (*O*-DMA) by normal flora of the gut. However, *O*-DMA is produced by a different group of normal flora [9]. *Eggerthella* sp. YY7918, *Slackia* sp. Strain NATTS [10,11] are among the colonic bacteria responsible for producing equol in humans. The variations of gut microflora among individuals resulted in only 30–50% and 80–90% of the population to produce equol and *O*-DMA, respectively [12]. This interindividual variation leads to inconsistent study outcomes related to the effects of soy in humans.

Equol was first discovered in 1932, where it was isolated from the urine of a pregnant horse [13]. In 1980, the unexpected discovery of a high amount of equol in rat urine and mammalian lignans, enterolactone, and enterodiol sparked an interest [14]. Equol was also discovered in sheep [15], hens [16], and dogs [17]. Equol [7-hydroxy-3-(49-hydroxyphenyl)-chroman], C_15_H_14_O_3_ has a molecular weight of 242.27 Daltons. It is a non-polar molecule, making it insoluble in solution [18] and is extremely acid-labile [19]. Equol has a chiral centre, which allows it to exist in two enantiomeric forms: *R*-equol and *S*-equol [18]. *S*-equol is the natural diastomer produced from intestinal metabolism. Adults consuming large amounts of soyfood were found to have high equol concentration in their urine [20]. Equol has a slower plasma clearance (6.85 L/h) compared to daidzein (17.5 L/h) [21]. Apart from its potential role in cancer, equol in health has been investigated in various studies. Equol has been shown to significantly reduce hot flushes in menopausal women [22], prevent a reduction in bone mineral density in middle-aged women [23], possibly help reduce the incidence of prostate cancer [24] and prevent the occurrence of breast cancer [25].

Setchell et al. introduced the terminology of “equol producer” and “non-equol producer” based on the individual’s plasma equol concentration [21]. Individuals with <40 nmol/L (10 µg/L) can be classified as “non-equol producer”, while >83 nmol/L (20 µg/L) are “equol producers”. An individual is also considered as an “equol producer” when urine equol concentration is >1000 nmol/L [26]. The percentage of equol producers reported in different population and countries ranges from 35% North American postmenopausal women [27], 45% Japanese male [24], 35% North American postmenopausal women [27], 51% of Irish Caucasian women [28] and 70.1% Koreans [29]. However, it is essential to note that plasma equol level can be measured in non-equol producers when high equol food sources, such as stinky tofu, cow milk, cheese, yoghurt, and egg, are consumed [30,31,32]. In vivo and in vitro studies show inconsistent results on the advantages or benefits of soy, due to factors such as the complexity of phytoestrogens and endogenous oestrogens. Of note, the equol producer status was not established in equol studies that involve the human population. The high interest in equol is due to the possibility that the efficacy of soy isoflavone depends on the ability of the individual to produce equol [33]. Hence, Setchell et al. (2002) highlighted that the inconsistent epidemiological findings of phytoestrogen studies might be due to the lack of emphasis on the subjects’ equol producer status in the population studied [21].

The role of equol as the key to soy food efficacy warrants further investigations [12]. One study suggested that soybean isoflavones can prevent the onset of prostate cancer either through mechanisms directly involving equol or daidzein’s transformation to equol [24]. Subsequently, it was thought that being an equol producer provides protective effect against prostate cancer. Surprisingly, equol was shown to have higher oestrogenicity with a 13-fold higher affinity to oestrogen receptors β (ER-β) than ER-α than its precursor daidzein [34]. Its presence in 30–50% of the population has led to the postulation that equol might have a role in hormone-dependent conditions [21]. The postulation is supported by a previous study that reported potential health benefits of equol, such as preventing breast cancer occurrence.

Breast cancer is the most frequent cancer among females, with an age-standardised rate of 29.1 per 100,000 populations. In 2012, 14.1 million new cancer cases were reported, and cancer has caused 8.2 million deaths or 14.6% of all human deaths [35]. A combination of surgery, radio- and chemotherapy remains the gold standard for cancer treatment. However, cancer therapies are still hindered by systemic toxicities, non-selective actions, and the emergence of drug-resistance tumour cell clones. Survival of patients with metastatic breast cancer has not improved significantly despite the development in early diagnosis and treatments of breast cancer [36,37]. Multidrug resistance remains the principal obstacle in treating metastatic breast cancer. This review aims to systematically review the literature on in vitro and in vivo evidence on the effects of equol on breast cancer.

## 2. Results

The search identified 1113 articles, and only 23 articles finally met the inclusion criteria. All papers were published between 1987 and 2020. There were 18 in vitro studies, two in vivo studies, and three in vitro and in vivo experiments. Based on all 23 studies included, four studies showed equol has neither oncogenic nor anticancer properties [38,39,40,41], six studies showed evidence of equol with oncogenic properties [42,43,44,45,46,47], while the other 13 studies concluded that equol has anticancer properties [48,49,50,51,52,53,54,55,56,57,58,59,60]. The studies included in this review involve the effect of equol and other isoflavones, such as daidzein and genistein. However, the emphasis was given to findings associated with equol and its effect on breast cancer that are summarised in the results. For in vivo studies, rodents, such as rats and mice, were used as the animal model. From this review, the results were categorised into three main sub-topics, which are (i) cancer properties of equol (ii) concentration variations of equol, and (iii) oncogenic pathways and mechanism.

### 2.1. Cancer Properties of Equol

Based on the results, each study’s conclusion is summarised according to evidence of equol cancer properties on breast cancer, oncogenic properties, anticancer, or no effect. Out of 13 publications that reported anticancer properties of equol, ten are in vitro studies, and two are in vivo studies, while one is of a mixed study of both in vivo and in vitro. Publication years range between 2006 and 2019.

#### 2.1.1. In Vitro Studies

Several commercially available cell lines, mainly MCF-7, MDA-MB-453, -468, -231, SKBr-2, and HS578t, were used to evaluate the effect of equol on breast cancer cells. Besides MCF-7 cells, the effect of equol was also measured in ErbB2 positive cells (MDA-MB-453) and triple-negative breast cancer (TNBC) cell lines (ER-, PR-, ErbB2-), which are MDA-MB-468, MDA-MB-231, and HS578t. Interestingly, equol in the in vitro TNBC model was determined in two main subtypes: Basal-like subtype (MDA-MB468) and claudin-low (HS578t and MDA-MB-231) breast cancer cell lines. Out of the ten in vitro studies that reported anticancer properties, six studies utilised MDA-MB-231 cells [49,51,54,55,57,58], two studies utilised MDA-MB-453 cells [50,53]. Other studies also used MCF-10A cells [57,58], MDA-MB-468 cells [52], and T47D cells [54].

#### 2.1.2. MCF-7 Cells

Out of the ten in vitro studies that reported anticancer properties, seven studies published from 2006 to 2019 used MCF-7 cells [49,51,52,53,56,57,58]. Two other publications by Welsons et al. in 1987 [43] and Sathyamoorthy et al. in 1997 [44] used MCF-7 cells; however, reported oncogenic properties of equol. Two of the studies that reported on anticancer properties of equol using MCF-7 cells and *S*-equol observed different action mechanisms in their experiments. Zhang et al., 2019 reported that *S*-equol inhibits proliferation and promotes apoptosis of MCF-7 cells via regulating miR-10a-5p and PI3K/AKT pathway resulting in an increased level of apoptotic protein, such as cleaved caspase-9 and caspase-3 [51]. However, Bosviel et al., 2011, who also used *S*-equol and MCF-7 cells, reported a significant decrease in the cytosine phosphate guanine islands’ methylation in the promoters of BRCA1 and BRCA2. This was observed following 2 µM of *S*-equol treatment for three weeks in MCF-7 cells [57]. Hence, an increase in BRCA1 and BRCA2 proteins was also observed following the said treatment. Significant chemosensitisation was observed in MCF-7 cells treated with phytoestrogens and not MDA-MB-231 cells [49].

Similarly, Magee et al. had also explored daidzein and *S*-equol and racemic equol [58]. The racemic equol was determined to contain a mixture of *R*- and *S*-equol at a ratio of 1:1. The researchers found that *S*-equol was unable to prevent DNA damage in MCF-7 and MCF-10A upon exposure of HNE, menadione, or BPDE. Both daidzein and racemic equol stimulated MCF-7 growth at low concentration. Anticancer properties were not demonstrated well in MCF-7 cells compared to MDA-MB-231, which will be discussed in the following paragraphs. While Rigalli et al. explored daidzein in addition to *R*-equol and *S*-equol, another study had used genistein instead [52]. However, the researchers did not specify the type of equol used in the study. This study reported that the cytotoxic effect of genistein was significantly enhanced in the presence of equol in MCF-7 cells. However, no synergistic effect of equol and genistein was seen in cell cycle progression as equol arrested the G1 phase with the concomitant progression of cells at the G2/M phase. The combination of genistein and equol induced increase in cleaved PARP, downregulation of Bcl-xL, but with no changes in Bax.

Moreover, the phosphorylation of AKT and downstream mTOR were not affected by the treatment of genistein, equol or their combinations. Interestingly, one study explored the effects of equol and tamoxifen and discovered that equol enhances 4-hydroxy tamoxifen’s anti-proliferative effect in MCF-7 cells [56]. The combination of equol and 4-hydroxy tamoxifen activates caspase-mediated apoptosis. Further observation showed 4-hydroxy tamoxifen and equol induce a time-dependent reduction of Bcl-2:Bax ratio, promoting cytochrome c release and activating intrinsic apoptotic pathway. One study reported that equol at low concentration induced MCF-7 cell proliferation where increased proliferating cell nuclear antigen (PCNA) was observed in MCF-7 cells treated with low equol concentration [53]. The study also reported that equol did not induce cell cycle arrest at low concentration, but induced G2/M arrest at 100 µM.

#### 2.1.3. MDA-MB-231 Cells

Six studies used MDA-MB-231 cells. Five studies used these cells in addition to MCF-7 cells [49,51,57,58] or T47D cells [54]. However, only one study used MDA-MB-231 cells solely [55]. One of the studies using MDA-MB-231 cells discovered that both racemic and *S*-equol inhibited proliferation of MDA-MB-231, LNCaP, and LAPC-4 cells [58]. Also, daidzein, racemic, and *S*-equol inhibited invasion of MDA-MB-231 and prostate cancer cells. Rigalli et al. (2019) found that anticancer properties of both *S*-equol and *R*-equol were demonstrated by inhibition of metoxantrone efflux in BCRP+ (breast cancer resistance proteins) MDA-MB-231 cells [49]. This effect is also seen with daidzein, a common phytoestrogen frequently associated with soy products. The same study also reported on significant chemosensitisation observed in BCRP+ MDA-MB-231 cells toward mitoxantrone treated with phytoestrogens. For the study that solely used MDA-MB-231 cells without other accompanying cell lines, Magee et al. reported no significant inhibition of cell viability by *R*-equol, *S*-equol daidzein [55]. The study further elucidates that *R*-equol, *S*-equol, and daidzein induced a dose-dependent inhibitory effect on the invasive capacity of MDA-MB-231 cells. In addition, the downregulation of MMP-2 expression was also evident. However, no significant effects were observed on MMP-9, TIMP-1, or TIMP-2 expression. One study explored the effects of equol and radiation on breast cancer cells found that equol inhibited cell growth in both MDA-MB-231 and T47D in time and dose-dependent [54]. The study also reported that equol-treated irradiated cells significantly enhanced cell death compared to untreated irradiated MDA-MB-231 cells. Enhanced DNA breaks by irradiation were observed in equol treated MDA-MB-231 cells. Another study reported a significant decrease in the methylation of the cytosine phosphate guanine islands in the promoters of BRCA1 and BRCA2 following 2 µM of *S*-equol treatment for three weeks in MDA-MB-231 [57]. Moreover, there was an increase in BRCA1 and BRCA2 proteins following the treatment.

#### 2.1.4. MDA-MB-453 Cells

In a study by Choi and Kim, 2008, using both MDA-MB-453 cells and MCF-7 cells, significant inhibition of cell proliferation and induced cell cycle arrest and apoptosis in the ER-negative MDA-MB-453 cells was reported [53]. This study showed that equol significantly inhibited MDA-MB-453 cell proliferation in a dose- and time-dependent manner. However, equol at low concentration induced MCF-7 cell proliferation, where increased proliferating cell nuclear antigen (PCNA) was observed in MCF-7 cells treated with low equol concentration. Equol did not induce cell cycle arrest at low concentration, but induced G2/M arrest at 100 µM. This is accompanied by reduced G2/M proteins, such as CDK1, PCNA, and cyclin B, but not G1 regulator proteins, such as CDK2, CDK4, and cyclin D. Interestingly, equol affected cell cycle regulatory protein more significantly in MDA-MB-453 cells compared to MCF-7 cells. Equol dose-dependently increased p53, cytochrome *c,* and Bax and decreased Bcl-2 expression in MDA-MB-453 cells. Another study by Choi et al., 2009 reported anticancer properties of equol demonstrated by intrinsic apoptotic pathway via cytochrome *c* release as demonstrated in MDA-MB-453 cells [50]. A dose-dependent manner of decreased cell proliferation was observed after 48 h and 72 h treatment with equol, while an increase in apoptotic cells was observed after treatment with equol (50 μM and 100 μM) for 72 h. This is attributed to the increase in cytochrome *c* release, activation of caspase 3, 6, 7, 9, but not 8, and an increase in cleaved PARP after treatment with equol (50 μM and 100 μM) for 72 h.

#### 2.1.5. In Vivo Studies

Tumour animal models have been widely used in the development of new anticancer therapies. The in vivo studies on the effect of equol in breast cancer involve rodent models, chemically-induced tumours in Sprague-Dawley rats [48,60], and Balb/c athymic nude mice [39] inoculated with cancer cells or oestrogen hormone. The agent used to promote the formation of mammary tumours in Sprague-Dawley rats is 7-12-dimethylbenz(a)anthracene (DMBA), which stimulates the formation of mammary tumours by inducing point mutation, activating Ras gene, and perturbing redox balance [61]. DMBA-induced tumours were used to determine equol’s chemopreventive properties, whereby equol was administered before tumour induction [48], and the anticancer properties of equol were investigated when equol was administered upon tumour formation [60]. The tumours in Balb/c athymic-nude mice are induced either by placing pellets containing 2 mg 17β-oestradiol subcutaneously after ovariectomy [39,40,41] or inoculation of T547D:A18 cells and oestrogen-dependent human breast cancer cells [46]. Two studies with Sprague-Dawley rats demonstrated anticancer properties of equol [48,60]. The study by Brown et al. elucidated equol to significantly reduce tumour formation, increased tumour latency, and reduced tumour invasiveness compared with control and *S*-(−) equol. Both *R*-(+) and *S*-(−) equol showed significantly lower weight gain compared to control [48]. The study reported by Ma et al. found that ovariectomised rats fed with isoflavones and equol showed significant inhibitory effects on the development of DMBA-induced mammary tumours [60]. The study also concluded that equol has a higher antioxidant capacity than isoflavone treatment, although both isoflavones and equol intake significantly increased the Nrf2 expression.

#### 2.1.6. Mixed Studies

There are four mixed studies in which both in vitro and in vivo studies were reviewed. None of the four studies showed anticancer properties, one oncogenic, while another three showed no equol effects on breast cancer. The study that demonstrated oncogenic properties reported that genistein, daidzein, and equol do not inhibit T47D:A18/PKC (tamoxifen-resistant) autonomous cell proliferation, whereas only equol stimulates T47D/A18 (hormone-dependent) cell proliferation [46]. While equol induced ERE-luciferase activity in hormone-dependent cells (T47D:A18), equol and daidzein induced ERE-luciferase activity in a tamoxifen-resistant cell. Both equol and daidzein exhibit oestrogenic effect in tamoxifen-resistant cells, but this effect is reversed by antioestrogen. The authors then conclude that tamoxifen administration with either daidzein or genistein can produce tumours of greater size than with tamoxifen alone, which suggests that it is unsafe to consume isoflavones supplement with tamoxifen. Results that concluded equol has no effect on breast cancer showed no statistical difference between MCF7 and (±)-equol treatment groups on tumour surface area, cell proliferation (Ki-67 expression), and pS2 mRNA level [39]. One study which concluded equol does not affect breast cancer demonstrated that equol and genistein stimulated T47D cell proliferation similar to 17β-oestradiol, and this response was inhibited by tamoxifen [41]. However, no stimulation of MCF-7 tumour growth was observed with an increasing amount of equol. Neither was there any additive effect observed when genistein and equol were combined. Another study that concluded no effect by equol demonstrated that purified *S*-equol, daidzein, glycitein, genistein, and isoflavonoid mixture increased MCF-7-E10A cell proliferation compared to control. However, when compared to control, no statistical significance was found between *S*-equol and SE5-OH supplement in the time course of tumour size [40].

### 2.2. Concentration Variations of Equol

Based on the results, we make the following observations regarding equol types and their concentration used in the experiments. Out of 23 studies, four studies used only *S*-equol [38,40,51,57], and six studies used both *R*- and *S*-equol enantiomers [46,47,48,49,55,58], while thirteen other studies did not specify the equol enantiomer used. The majority of these 13 studies were published before 1995. Noticing that researchers selected a wide range of equol concentration, we grouped the concentration according to the type of equol enantiomer used. The *R*-equol concentration ranged from 0–50 µM, while *S*-equol concentration for all the studies ranged from (0–2063 µM). As for studies with non-specific equol enantiomer, the concentration used ranged from 0 to 100 µM. We further categorised the equol concentrations into three outcomes (anticancer, no effect, and oncogenic), as outlined in Table 1. Based on the equol concentration used in the studies, there is no definite pattern of low or high concentration that tend to possess more anticancer or oncogenic outcomes. Out of the 13 studies that demonstrated anticancer properties, a wide range of equol concentration ranging from 0–619.14 µM. However, studies with oncogenic outcomes showed a narrower range of equol concentration used by researchers, ranging from 0.001–25 µM. There is no standardised concentration range to be tested in in vitro or in vivo studies, making it more challenging to narrow down the most effective or definitive concentration range where equol is most effective.

### 2.3. Oncogenic Pathways and Mechanism

**Figure 1:**
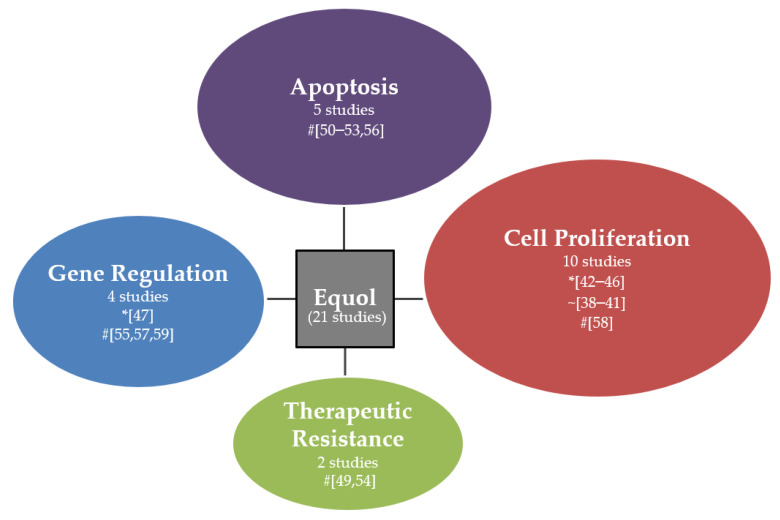
Pathways affected by equol. “#”: anticancer properties; “*”: oncogenic properties; “~”: no effect.

To review the oncogenic pathways and mechanism, we categorised the pathways affected by equol into cell proliferation, gene regulation, apoptosis, and therapeutic resistance, as shown in Figure 1. Two studies were not included in the figure as no specific pathways were the focus of the study. Ten studies are focusing on the cell proliferation pathway. From these ten studies, five reported equol to be oncogenic [42,43,44,45,46], and one study reported an anticancer outcome. The remaining four studies reported that equol had neither oncogenic nor anticancer properties. Equol activated oestrogen-dependent cell proliferation via several mechanisms, including MEK/ERK signalling [45]. The study by Magee et al. is the only study that reported equol to inhibit breast cancer cell proliferation [58]. In contrast to the study by Liu et al., the equol concentration used in the study by Magee et al. was five times higher than in Liu’s study. Hence, the higher concentration of equol used might explain equol’s dual role in regulating breast cancer cell proliferation, which is affected by the equol concentration. Then, five studies reported anticancer properties of equol, which involves inducing intrinsic apoptotic pathway [50], inhibiting expression of PI3K p110α and decreasing phosphorylated Akt level [51]. Equol increases the proapoptotic protein levels mainly Bax/Bcl-xl expression ratio [52] and p53 [53]. Equol was also shown to reduce the antiapoptotic protein Bcl-2/bax ratio and increase the release of cytochrome c [56]. Only four studies explored on gene regulation pathway. Equol was reported to transcriptionally regulate proteins involved in tumourigenesis by upregulating c-Myc via enhanced transcription of eIFgI [47]. In contrast, several other studies reported the anticancer properties of equol mainly mediated via reduced expression of MMP, which results in impaired metastasis characteristics in breast cancer cells, as well as enhanced demethylation of BRCA1 and BRCA2 promoters that reduces the risk of early onset of breast cancer [57]. On therapeutic resistance, only two studies were involved. Both studies reported equol’s anticancer properties, which was demonstrated by inhibition of breast cancer resistance protein [49], apoptosis, and anti-proliferative effects on radioresistant cells [54].

## 3. Discussion

This review aims to compare the effects of equol on breast cancer cells based on factors, such as range of equol concentrations and mechanism of action based on in vitro and in vivo studies. Lack of standardised equol concentration and inconsistent selection of equol enantiomers, *R*-equol, or *S*-equol used in both in vitro and in vivo studies limits the comparison on the outcome between studies. Although the oncogenic properties of equol are demonstrated by five studies that used equol concentrations that range from 0–25 µM [42,43,44,46,47], it is not conclusive if a higher concentration of more than 25 µM will result in anticancer properties. Out of the 13 studies demonstrating anticancer properties, the equol concentration used in the experiments included a range lower than 25 µM and extended to 350 µM. *S*-equol is a daidzein metabolite produced in vivo, while *R*-equol can be synthetically produced [34]. Binding preference for *S*-equol and *R*-equol is ER-β and ER-α, respectively [34]. Studies that employ only *S*-equol provides information on the effects of the naturally produced metabolite in vivo, which is of great interest as it applies to humans who are 30–50% equol producers and animals. Whereas, studies that investigated both enantiomers provide information on efficacy and compare the effects of *R*-equol. Production of *S*-equol from daidzein is associated with several intestinal bacteria strains. A recent study reports a detailed list of 29 intestinal bacterial strains that biotransform isoflavones to equol [18]. Among the bacteria found in the human intestine are *Adlercreutzia equolifaciens* [62] Clostridium like bacterium and *Escherichia coli sp* [63], *Eggerthella sp* [64], and *Lactobacillus mucosae* [65].

Majority of the in vitro studies demonstrating anticancer effects involved MCF-7 breast cancer cells. Other cell lines include MCF-7, MDA-MB231, MDA-MB453, MDA-MB268, and Hs578t cells. MCF-7 cell is classified as luminal A subtype of breast cancer, and it is largely used as the in vitro oestrogen-sensitive breast cancer model [66]. MCF-7 cells were reported to positively express oestrogen receptor (ER+) and progesterone (PR+), but not human epidermal growth factor receptor 2 (ErbB2-) [67]. The overexpression of ErbB2 leads to increased breast cancer metastasis [68]. However, the overexpression of this receptor allows the development of ErbB2-targeting therapeutic agents, such as trastuzumab, which was used for the past two decades in breast cancer therapy [69]. Triple-negative breast cancers (TNBCs) represent 15% of breast carcinomas and frequently have a poor prognosis [70]. TNBC comprises a heterogeneous group of tumours, and recently using the probabilistic graphical model, TNBCs are molecularly classified into claudin-low, claudin high, basal-like, and luminal androgen subtype [71]. The basal-like subtype shows similar gene expression of the normal breast cells’ basal-myoepithelial layer that demonstrates enhanced cell cycle and cell division rate [72,73]. This subtype is supported by increased expression of Ki677, which is strongly associated with aggressive tumour growth and cell proliferation, and decreased tissue differentiation [74]. TNBC cell lines of the claudin-low subtype were reported to express a negligible level of epithelial cell-cell adhesion proteins and highly express epithelial-mesenchymal transition genes [75]. This subtype is characterised by reduced cell proliferation rate, genomic stability, and mutational burden with marked immune and stromal cell infiltration [76]. Nude athymic mice allow routine and efficient transplantation and formation of tumours, due to the T-cell deficient property of the mice [77]. The heterotopic and orthotopic model utilised to induce tumour is commonly used as a tool to evaluate the oncogenic or chemopreventive properties of an agent at the preclinical phase [78]. From the studies reviewed, we conclude that equol can act on various pathways. In studies that investigated both equol enantiomers, the results between *R*- and *S*-equol are similar in several pathways, such as gene regulation [55], cell proliferation [46,58], and therapeutic resistance [49]. This suggests that *R*-equol, which can be synthesised, is as potent as the naturally produced *S*-equol. This triggered an interesting possibility of *S*-equol being used as a supplement in various medical areas, such as brain development. From this review, more in vitro evidence of equol has anticancer properties than there are of equol demonstrating oncogenic activities. However, a definitive link between the effective concentration range of equol and its beneficial effects has been difficult to prove. This could, in part, be explained by several factors associated with equol. Firstly, it is due to the dual nature of equol that can act both as agonist and antagonist [21]. To date, no established factors can be ascertained to trigger its role as agonist or otherwise. *S*-equol is an oestrogen agonist [79]. There is a likelihood that more in vivo studies are necessary to confirm the effects of equol. Apart from studies on the effects of equol on breast cancer, many studies have investigated how equol affects other systems. Equol is associated with reduced risk of coronary heart disease [80], reduction of hot flushes during menopause [81], and reduced risk of developing breast cancer [82]. As equol can cross the blood-brain barrier, studies have also explored how equol affects the brain. Equol is responsible for cell proliferation in breast cancers and is also found to induce cell proliferation mechanisms in astrocytes [83] and produce anti-depressant effects [84]. Though the mechanisms are not clear, there is growing evidence of neuroprotective effects of dietary equol and isoflavones [85]. Due to the many potential health benefits of equol, researchers are interested in knowing if the conversion of non-equol producers into equol producers is possible. The conversion of non-equol producers to produce equol has been proven successful by Tanaka et al., 2009 upon reporting that 2 out of 10 non-equol producers were converted to equol producers after receiving 60 mg of soy supplements daily for three months. However, more studies reported failed conversion to equol producers. Another failed attempt in converting non-equol producer to equol producer involved the consumption of probiotics, such as *Lactobacillus acidophilus* and *Bifidobacterium longum* for two months [86]. However, consumption of soybeans and green tea is reported to increase equol production [87]. Limitations of this systematic review include the fact that there is limited information on the type of equol being used and the non-uniformed concentrations tested. For studies with unspecified types of *R*- or *S*-equol, the effects may be under or overestimated.

## 4. Methods

### Search Strategy and Selection Criteria

**Figure 2:**
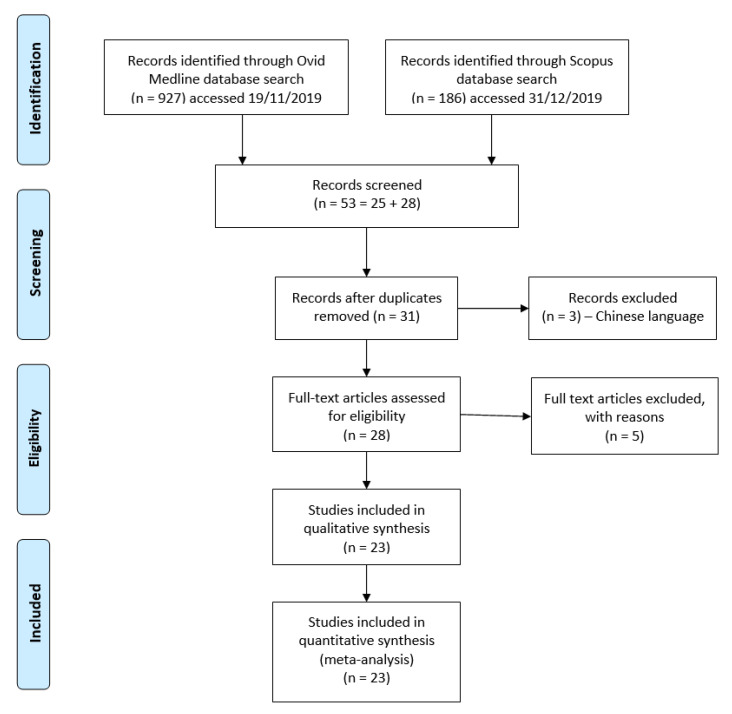
Flowchart for identification of relevant studies for a systematic review.

Databases searched for potentially relevant studies include the Ovid MEDLINE database (1990–2019) accessed on 19th November 2019, Scopus database (1970–2019) accessed on 31st December 2019. Databases were searched for English-language articles using the relevant keywords yielded 1113 potentially relevant studies. All English-language studies of equol (exposure variable) on breast cancer outcomes (dependent variable) were considered for inclusion. Keywords used are ‘equol,’ and ‘breast cancer’ OR ‘breast carcinoma’ OR ‘breast neoplasia’ OR ‘breast malignancy’ OR ‘mammary cancer’ OR ‘mammary carcinoma’ OR ‘mammary tumour’ OR ‘ mammary neoplasia’ OR ‘ mammary malignancy.’ After the manual screening, based on types of articles and titles, 56 articles were shortlisted. Following the removal of duplicates, there were 31 articles. Three studies in the Chinese language were excluded. Out of 28 full-text articles assessed, four articles involving human studies, and one review article were also excluded. Finally, 23 studies met the inclusion criteria, as shown in the PRISMA flow diagram (Figure 2). Search and identification of studies for inclusion was performed simultaneously by all authors, whereas data abstraction and quality assessment were performed independently by each author using a data extraction form. We resolved discrepancies following independent data abstractions and quality assessments until there was a 100% agreement. Studies included publications ranging from the year 1987–2020 (Table 2).

## 5. Conclusions

In conclusion, this review reports more evidence to support that equol has more anticancer than oncogenic properties. However, it is inconclusive at which concentration is equol, demonstrating anticancer properties or oncogenicity. We would like to recommend that future studies to establish an IC50, as it is a more specific indicator of a significant concentration range for the outcome measured. Equol acts on multiple pathways and in various types of breast cancer cells. However, it is uncertain on which pathway does equol work best to produce anticancer effects. More research should focus on gene regulation pathways to better understand the mechanism of action of equol.

## Figures and Tables

**Table 1:** Comparison of equol concentrations used in in vitro and in vivo studies.

Anticancer (13 Studies)		No Effect (4 Studies)		Oncogenic (6 Studies)	
Ref, Year	Equol Concentration	Ref, Year	Equol Concentration	Ref, Year	Equol Concentration
[58] 2006	0–50 µM *R*-equol and *S*-equol	[40] 2011	* 250 ppm–500 ppm (1031.9–2063.8 µM) *S*-equol	[43] 1987	0.1–1 µM
[55] 2014	0–50 µM *R*-equol and *S*-equol	[39] 2006	* 0–100 µM	[44] 1997	0.001–1 µM
[53] 2008	0–100 µM *R*-equol and *S*-equol	[38] 2018	1 µM *S*-equol	[44] 1997	0.001–1 µM
[50] 2009	0–100 µM *R*-equol and *S*-equol	[41] 2020	* 0–100 µM 1.72–4.42 µM	[46] 2007	0.1 µM *R*-equol and *S*-equol
[51] 2019	0–150 µg/mL (0–619.14 µM) *S*-equol			[42] 2009	10 µM
[48] 2010	* 36.8 0 ± 8.4 ng/mL (0.15 µM ± 0.03 µM) of *R*-equol 13.9 ± 2.3 ng/mL (0.05 µM ± 0.002 µM) of *S*-equol			[45] 2010	10 µM
[59] 2006	1 µM			[47] 2015	25 µM
[57] 2012	1 µM (*S*-equol)				
[54] 2015	50 µM				
[52] 2017	50 µM				
[56] 2013	100 µM				
[60] 2014	* 100–400 mg/kg				
[49] 2019	10 µM (*S*-equol) 10 µM (*R*-equol)				

* in vivo studies.

**Table 2:** Characteristics of relevant studies.

Ref	Study Population	Study Design	Type of Intervention	Conclusion	Cancer Properties
[38]	In vitro	MCF-7	*S*-equol Daidzein	Equol does not stimulate the generation of cancer cells.	No effect
[48]	In vivo	Tumour-induced Sprague-Dawley rats	Group 1: AIN93G diet Group 2: 250 mg/kg *R*-(−)equol Group 1: 250 mg/kg *S*-(−)equol	*R*-(+) is chemopreventive and increases tumour latency in the animal model. *S*-(−) equol has no chemopreventive or stimulatory effect.	Anticancer
[42]	In vitro	HeLa HepG2 MCF-7	Genistein Daidzein Equol	Erα transcriptional activation by isoflavones (genistein and daidzein) and equol is mediated through AF-1 and have similar properties to oestradiol on Erα expression and MCF-7 cell proliferation.	Oncogenic
[50]	In vitro	MDA-MB-453	Equol	Equol induced intrinsic apoptotic pathway via cytochrome c release and caspase 9 in human breast cancer MDA-MB-453 cells.	Anticancer
[39]	In vitro	MCF-7	In vitro (±)-equol Daidzein	(±)-equol stimulates ER-regulated MCF-7 cells growth, but daidzein and (±)-equol is not the active isoflavones that affect breast cancer growth in athymic mouse-MCF-7 xenograft preclinical model for postmenopausal oestrogen-dependent breast cancer.	No effect
	In vivo	Athymic BALB/c mice	In vivo (±)-equol 250 ppm Group 7 (±)-equol 500 ppm Group 8 (±)-equol 1000 ppm		
[51]	In vitro	MCF-7 Control- MCF-10A Others: MDA-MB-231 SKBr-3	*S*-equol	*S*-equol upregulates the expression of miR-10a-5p in MCF-7 cells to inhibit the activation of the PI3K/Akt pathway and subsequently regulate the downstream apoptotic cascade.	Anticancer
[49]	In vitro	MCF-7 MDA-MB-231	Daidzein *R*-equol *S*-equol	Daidzein, *S*-equol, and *R*-equol treated cells showed a significant decrease in doxorubicin and metoxantrone efflux by potent inhibition of breast cancer resistance protein (BCRP).	Anticancer
[43]	In vitro	MCF-7 T47D	Enterolactone Equol	Equol stimulates in vitro breast cancer cell proliferation.	Oncogenic
[44]	In vitro	MCF-7	Equol Daidzein	Equol has a higher affinity for the ER in breast cancer cells compared to daidzein.	Oncogenic
[52]	In vitro	MCF-7 Others: MDA-MB-468 SKBr-3	Genistein Equol Genistein + equol	The synergistic growth-inhibitory mechanism of genistein in a combination of equol is postulated to be mediated by modulation of Bax and Bcl-xL expressions.	Anticancer
[53]	In vitro	MCF-7 MDA-MB-453	Equol	Equol significantly inhibited cell proliferation and induced cell cycle arrest and apoptosis in ER-negative MDA-MB-435 cells.	Anticancer
[54]	In vitro	MDA-MB-231 T47D	Equol Radiation + equol	Equol exhibits anticancer properties: To inhibit proliferation, induce apoptosis, and enhanced radiosensitivity on both oestrogen-positive and -negative breast cancer cells.	Anticancer
[55]	In vitro	MDA-MB-231	Daidzein *R*-equol *S*-equol	Daidzein, *R*-equol, and *S*-equol inhibited the invasion of MDA-MB-231 human breast cancer cells, in part, via down-regulation of MMP-2.	Anticancer
[56]	In vitro	MCF-7	Equol Tamoxifen Equol + tamoxifen	Equol induces MCF-7 cell apoptosis and enhances tamoxifen’s pro-apoptotic effect via activation of the intrinsic apoptotic pathway.	Anticancer
[57]	In vitro	MDA-MB-231 MCF-7 MCF-10A	*S*-equol	*S*-equol has a demethylating effect on cytosine phosphate guanine islands in the promoters of BRCA1 and BRCA2 genes.	Anticancer
[40]	In vitro	MCF-7 MCF-7-E10	In vitro *S*-equol Daidzein Glycitein Genistein Isoflavonoids mixture	Purified *S*-equol, SE5-OH, and genistein do not stimulate oestrogen-dependent or-independent MCF-7 cell proliferation at a concentration less than or equal to 500 ppm.	No effect
	In vivo	Athymic BALB/c mice	Group 1 AIN-93G Group 2 *S*-equol 250 ppm Group 3 *S*-equol 500 ppm Group 4 Genistein 250 ppm Group 5 Genistein 500 ppm Group 4 Natural *S*-equol supplement (SE5-OH) 250 ppm Group 4 Natural *S*-equol supplement (SE5-OH) 500 ppm		
[45]	In vitro	MCF-7 MDA-MB-231	Genistein (±)-equol	Low concentration of genistein and equol stimulate cell growth and cell cycle progression via ER-mediated transcription and activation of ERK1/2.	Oncogenic
[46]	In vitro	T47D:A18/PKC (tamoxifen resistant) T47D/A18 (hormone-dependent)	(*R*,*S*)-equol Daidzein Genistein	Simultaneous consumption of isoflavone supplements with tamoxifen may not be safe.	Oncogenic
	In vivo	Athymic BALB/c mice	Group 1 Control Group 2 17β-oestradiol Group 3 Tamoxifen Group 4 Genistein Group 5 Genistein + tamoxifen Group 6 Daidzein Group 7 Daidzein + tamoxifen		
[58]	In vitro	MCF-7 MDA-MB-231, MCF-10A LNCap LAPC-4 PC-3	Daidzein *S*-equol *R*-equol	Racemic and *S*-equol showed equipotent biological effects on breast and prostate cancer cell proliferation and invasion in vitro.	Anticancer
[59]	In vitro	MCF-7 Caco-2	Genistein Equol	17β-oestradiol and genistein at physiological doses upregulate CYP27B1 (vitamin D activation) and downregulate CYP24 (vitamin D hydroxylase catabolism).	Anticancer
[60]	In vivo	Ovariectomised Sprague Dawley	Group 1 AIN-93G Group 2 AIN-93G + 100 mg/kg isoflavones Group 3 AIN-93G + 500 mg/kg isoflavones Group 4 AIN-93G + 1000 mg/kg isoflavones Group 5 AIN-93G + 100 mg/kg equol Group 6 AIN-93G + 200 mg/kg equol Group 7 AIN-93G + 400 mg/kg equol Group 8 AIN-93G + 2.5 mg/kg stilboestrol	Isoflavones and equol intake significantly inhibited the development of postmenopausal mammary tumours by antioxidant and oestrogenic activities.	Anticancer
[49]	In vitro	MCF-7 T47D	Genistein (±)-Equol	Dietary equol intake did not alter the oestrogen-dependent tumour growth in either T47D or MCF-7 models in long term studies.	No effect
	In vivo	Ovariectomised athymic BALB/c nude mice	Mice injected with T47D Group 1 Negative Control (AIN-93G) Group 2 Positive control (17B-oestradiol) Group 3 AIN-93G + 250 ppm equol Group 4 AIN-93G + 750 ppm equol Mice injected with MCF-7 Group 1 Negative Control (AIN-93G) Group 2 Positive control (17B-oestradiol) Group 3 AIN-93G + 250 ppm equol Group 4 AIN-93G + 750 ppm equol Group 5 500 ppm genistein Group 6 500 ppm genistein + 250 ppm equol Group 7 500 ppm genistein + 500 ppm equol Group 8 500 ppm genistein + 1000 ppm equol		
[47]	In vitro	MDA-MB-435 (ER-) Hs578t (ER-) MCF-10A	(*R*,*S*)-equol	Equol possesses pro-cancer properties and may influence cancer potential via the upregulation of c-Myc transcription, leading to c-Myc dependent and -independent eIF4G-mediated translation initiation of oncogenes increased cancer cell survival.	Oncogenic
